# Supplementary material for: The Effect of the COVID-19 Pandemic on the Assessment of Sexual Life—Repeated Cross-Sectional Surveys among Polish Adults in 2017, 2020 and 2021
Source: Int J Environ Res Public Health. 2022 Mar 30;19(7):4110. doi: 10.3390/ijerph19074110 (PMC8998642; doi:10.3390/ijerph19074110)
Supplement: Supplementary file 1 [file ijerph-19-04110-s001.zip › ijerph-1638719-supplementary.pdf]

International Journal of
Environmental Research and Public Health

MDPI

Table S1. Mean score per item* according to sexual life assessment profile** and year of data collection.

Item	2017 - Sexual life assessment profile									
	1 (*N*=167)		2 (*N*=292)		3 (*N*=244)		4 (*N*=377)		5 (*N*=962)	
	Mean	SD	Mean	SD	Mean	SD	Mean	SD	Mean	SD
What is the role of sex in your life at present?	0.63	0.64	2.53	0.65	0.96	0.60	1.99	0.39	3.29	0.48
How satisfied are you with your sex life over the past 2-3 months?	1.12	0.79	1.86	0.71	2.10	0.58	2.89	0.51	3.44	0.52
How do you assess the level of your sexual needs over the past 2-3 months?	1.25	1.00	2.33	0.64	0.98	0.72	1.91	0.50	2.62	0.67
Have your sexual needs over the past 2-3 months been fulfilled?	0.68	0.58	1.53	0.67	2.50	0.61	3.17	0.48	3.44	0.53
Item	2020 - Sexual life assessment profile									
	1 (*N*=196)		2 (*N*=380)		3 (*N*=263)		4 (*N*=752)		5 (*N*=827)	
	Mean	SD	Mean	SD	Mean	SD	Mean	SD	Mean	SD
What is the role of sex in your life at present?	0.90	0.71	2.77	0.79	1.23	0.68	2.52	0.57	3.46	0.52
How satisfied are you with your sex life over the past 2-3 months?	0.68	0.73	1.46	0.81	1.99	0.64	2.92	0.49	3.70	0.49
How do you assess the level of your sexual needs over the past 2-3 months?	1.24	1.06	2.61	0.79	0.93	0.72	1.99	0.43	2.87	0.75
Have your sexual needs over the past 2-3 months been fulfilled?	0.46	0.57	1.33	0.80	2.40	0.70	3.11	0.50	3.61	0.54
Item	2021 - Sexual life assessment profile									
	1 (*N*=180)		2 (*N*=324)		3 (*N*=225)		4 (*N*=611)		5 (*N*=640)	
	Mean	SD	Mean	SD	Mean	SD	Mean	SD	Mean	SD
What is the role of sex in your life at present?	0.77	0.66	2.75	0.81	1.12	0.63	2.42	0.63	3.43	0.54
How satisfied are you with your sex life over the past 2-3 months?	0.78	0.85	1.23	0.85	2.07	0.70	2.95	0.53	3.69	0.50
How do you assess the level of your sexual needs over the past 2-3 months?	1.36	1.12	2.68	0.74	0.93	0.75	2.00	0.46	2.87	0.72
Have your sexual needs over the past 2-3 months been fulfilled?	0.52	0.58	1.24	0.88	2.56	0.78	3.20	0.55	3.61	0.53

*responses ranged 1 to 4 (most positive one); ** profiles determined using 2017 data, as described in the paper.

Table S2. Ordinal regression models.

Variable	Model 1			Model 2		
	Estimates	SE	*p*	Estimates	SE	*p*
PROFILE 1 - worst	-3.94	0.18	<0.001	-3.80	0.17	<0.001
PROFILE 2	-2.71	0.18	<0.001	-2.55	0.17	<0.001
PROFILE 3	-2.13	0.18	<0.001	-2.02	0.16	<0.001
PROFILE 4	-1.09	0.17	<0.001	-0.80	0.16	<0.001
PROFILE 5 - best (ref.)						
Age (cont.)	-0.01	0.00	<0.001	-0.01	0.00	<0.001
Gender						
Female	-0.18	0.04	<0.001	-0.21	0.04	<0.001
Level of education						

lower than secondary	0.06	0.05	0.271	0.15	0.05	0.002
secondary	0.04	0.06	0.463	0.10	0.06	0.082
Place of living						
rural areas	-0.09	0.07	0.231	0.02	0.07	0.721
smaller towns	-0.03	0.07	0.669	0.05	0.07	0.488
Employment status						
working	0.09	0.05	0.045	0.00	0.04	0.948
Family material status						
low	-0.15	0.07	0.025	-0.06	0.06	0.357
average	-0.13	0.05	0.012	-0.08	0.05	0.116
Religious beliefs						
Believers	0.04	0.07	0.513	0.09	0.06	0.112
Status of relationship						
single	-0.61	0.06	<0.001	-0.59	0.06	<0.001
informal union	0.20	0.06	<0.001	0.15	0.06	0.009
Sexual orientation						
heterosexual	-0.16	0.09	0.076	0.02	0.08	0.775
Self-rated sexual performance						
poor	-1.63	0.09	<0.001	-1.95	0.08	<0.001
average	-1.27	0.07	<0.001	-1.40	0.07	<0.001
good	-0.79	0.06	<0.001	-0.89	0.06	<0.001
Last sexual intercourse						
this week	0.87	0.05	<0.001	0.74	0.05	<0.001
Difficulties hindering sexual activity						
none	0.07	0.04	0.114	0.11	0.04	0.011
Chronic condition						
no	0.12	0.05	0.014	0.10	0.04	0.033
Survey						
2020	-0.22	0.05	<0.001	-0.14	0.04	0.001
Nagelkerke R-sq.		0.400			0.388	

SE – standard error; Survey: 2017 as reference in Model 1; 2021 in Model 2; Other reference categories: profile 5; male, higher education; large cities; not working (student, unemployed, other); high material status; disbelievers; married; non-heterosexual; very good sexual performance; last intercourse not this week; at least one difficulty hindering sexual activity, chronically ill.
